# Causes of discontinuity of blood donation among donors in Shiraz, Iran: cross-sectional study

**DOI:** 10.1590/S1516-31802010000500006

**Published:** 2010-09-02

**Authors:** Leila Kasraian

**Affiliations:** I IMD. Community Medicine Specialist and Education and Research Manager, Shiraz Blood Transfusion Organization, Shiraz, Iran

**Keywords:** Blood, Blood donors, Blood banks, Supply and distribution, Patient selection, Sangre, Donantes de sangre, Bancos de sangre, Suministro y distribución, Selección de paciente

## Abstract

**CONTEXT AND OBJECTIVE:**

The adequacy of blood depends on blood donation rates and numbers of blood donors. To prepare adequate blood supplies, it is essential to investigate the barriers and factors that stop individuals from donating. This study aimed to identify the causes of lapsed donation at our center.

**DESIGN AND SETTING:**

Cross-sectional study of volunteer blood donors in Shiraz, Iran.

**METHODS:**

We selected 850 donors who had donated between January 1, 2005 and June 1, 2005, but had not donated again by June 2008. The participants were recruited by letter and telephone, and were interviewed using a specially designed questionnaire that contained items on demographic characteristics, donor motivations and reasons for not returning to donate. We used the chi-square test to identify associations between lapsed donor characteristics and reasons for declining to donate.

**RESULTS:**

The greatest motivation for donation was altruism. The most frequent reasons for lapsed donation were lack of time because of work and self-exclusion for medical reasons. Among first-time donors, the most frequent reasons were unsuitability for donation and lack of time.

**CONCLUSIONS:**

The reasons for not returning to donate are varied and may correlate with demographic characteristics. In this study, the main reason for not returning was lack of time. Changing donation hours so that donors can donate after work, providing mobile teams at workplaces, and shortening the duration of the donation process may help increase repeat donation rates.

## RESUMO

**CONTEXTO Y OBJETIVO:**

La idoneidad de la sangre y los productos sanguíneos depende de las tasas de donación y el número de donantes. Para preparar un suministro suficiente es esencial conocer las barreras y los factores que llevan a los individuos a dejar de donar. Hemos diseñado un estudio tipo encuesta para identificar las causes de la dejación de donar en nuestro centro.

**DISEÑO Y EMPLAZAMIENTO:**

Estudio transversal de los donantes de sangre voluntarios en Shiraz (Irán).

**MÉTODOS:**

Hemos seleccionado 850 donantes históricos que donaron entre el 1 de enero del 2005 y el 1 de junio del 2005, pero que no habían vuelto a donar para junio del 2008. Los participantes fueron reclutados por escrito (carta) y por teléfono, y fueron entrevistados con un cuestionario diseñado específicamente para este estudio y que contenía ítems sobre las características demográficas, las motivaciones del donante y las razones por las que no había vuelto a donar. Usamos la prueba de la ji-cuadrado para identificar las asociaciones entre la dejación de donar y las razones por dejar de donar.

**RESULTADOS:**

La motivación más frecuente para donar fue el altruismo. Las razónes más frecuentes para dejar de donar fue la falta de tiempo por motivos laborales, y la auto-exclusión por razones médicas. En los donantes primerizos, las razones más frecuentes fueron la no-idoneidad para donar y la falta de tiempo por motivos laborales.

**CONCLUSIONS:**

Las razones que llevan a la dejación de donar son diversas y pueden estar relacionadas con las características demográficas. In este estudio la razón principal fue la falta de tiempo por motivos laborales. Un cambio del horario de donación para permitir que los donantes puedan acudir después de la jornada laboral, los equipos móviles en los lugares de trabajo, y la reducción de la duración del proceso de donación pueden ayudar a aumentar la tasa de donación repetida.

## INTRODUCTION

Demands on blood supplies are increasing due to increasing life expectancy, aging populations, increasing donor losses due to new and sensitive screening tests and new exclusion criteria, confidential self-exclusion, and efforts toward improved blood safety. The adequacy of blood and blood products depends on the blood donation rate and numbers of blood donors.^1-8^ To ensure adequate blood supplies, programs to recruit first-time blood donors to compensate for blood donors who stop donating due to age, illness, donor screening results or other screening tests need to be planned.^9,10^

In other words, repeat donors play an important role in ensuring adequate blood supplies. Recruitment of repeat donors is easier because of better cooperation^11^ and fewer donor reactions.^12^ Moreover, the cost-benefit ratio of repeat donation is better, and these donors require less education to be convinced to return.^13^ To improve blood safety, the rate of repeat donors should be increased. Some studies have shown that the rate of blood-transmissible diseases was lower with blood from repeat donors, and that these donors more often donated for altruistic reasons and felt greater responsibility toward recipients.^14-21^

Although a large number of people are eligible to donate, only a few individuals donate and only a small number of them donate blood regularly.^13,22,23^ In a study in the United States, out of 60% of the population that was eligible to donate, only 5% actually donated.^13^ Another study showed that only 6% of first-time donors returned for donation regularly, and 62% of them never donated again.^11,24^ Another study in the United States found that about 19% of blood donors were first-time donors; however, 89% of the women and 63% of the men did not donate again.^25^ The number and the return rate of repeat donors were found to be an important issue.^12,23,26-28^ A study in Australia showed that if the donor return rate decreased from 88% to 85% over a two-year period, the number of first-time donors would be increased to 33%.^22^

A previous study showed that a higher donor return rate was influenced by having information about patients’ needs, having good experiences with previous donations and receiving assurance from the blood center.^29^ A lower rate was correlated with medical unfitness for donation, fear of side effects, not having enough time to donate, inaccessibility of donor centers, unsuitability of donation hours and having bad experiences with previous donations.^29-36^

## OBJECTIVE

In order to ensure adequate blood supplies, it is essential to have information about barriers and factors that impede donation.^11,13,22,24^ We designed the present study to investigate the causes of discontinuity of blood donation among donors in Shiraz, Iran.

## METHODS

In this cross-sectional study of blood donors in Shiraz, Iran, the sample size was calculated as 368 individuals, in accordance with previous studies,^11,24^ assuming a 60% rate of lapsed donation. To increase the power of the study, we selected 850 donors among those who donated between January 1, 2005, and June 1, 2005, but had not donated again by June 2008. All the donors were volunteers and they ranged in age from 17 to 65 years. A letter was sent to all 850 individuals to invite them to participate in a research project at the donation center. After two months, about 320 donors had responded to the invitation. The non-responders were contacted by telephone and invited again to report to the center, with assurances that there were no problems with their donated blood and no concerns about their health. In response, about 340 additional participants were recruited. Volunteers who indicated that they had donated blood elsewhere between June 1, 2005, and the time of the study were excluded from the first sample. All the participants included were interviewed using a specially-designed questionnaire that contained items about demographic characteristics, donor motivations and the reasons for not returning to donate blood.

The demographic variables recorded were age, sex, marital status, education level and donor status (first-time or repeat donor).

We used the chi-square test to identify associations between the demographic characteristics of lapsed blood donors and the reasons for declining to donate. Statistical analyses were done using the Statistical Package for the Social Sciences (SPSS) software, version 14.

## RESULTS

We invited 850 lapsed donors to take part in this study, and 660 of them agreed to participate (response rate of 77.7%). The volunteers who indicated that they had donated blood elsewhere between June 1, 2005 and the time of the study were excluded from the study and did not enter the final sample. The demographic characteristics of the sample are shown in Table 1. The response rate was higher among repeat donors, more highly educated donors and older donors (P < 0.05). Table 2 summarizes the motivations for giving blood that donors mentioned. The most common motivation for donation was altruism. The domains and deterrent factors in their decision not to return to donate blood are given in Table 3.

**Table 1.** Demographic characteristics of participants in this study

Age (years)	32.6 ± 9.3	
Gender	Men	531 (80.4%)
	Women	129 (19.6%)
Marital status	Married	459 (69.5%)
	Single	201 (30.5%)
Education	Less than secondary school	408 (61.8%)
	Secondary school or higher	252 (38.2%)
Donor status	Repeat donors	362 (54.9%)
	First-time donors	298 (45.1%)

**Table 2.** Blood donor motivations

Donor motivation	Number
Altruism	(73.6%) 486
Positive effect of donation on health	(18.2%) 120
Blood tests free of charge	(5.1%) 34
Religious reasons	(0.7%) 5
Family need	(1.8%) 12
Curiosity about donation	(0.6%) 3

**Table 3.** Domains and deterrent factors for not donating blood

Domain	Factor
No access to blood donation center	Donation centers hard to reach
Staff factors	Staff skills were poor. Bad treatment by staff and physician
Previous bad donation experience	Previous bad experience because of donor reaction or weakness after donation
Long wait to donate	Donation process was lengthy, not enough time
Travel inconvenience	Moved out of town, or job relocated out of town
Fear	Fear of needles, dislike of the sight of blood, fear of getting diseases due to donation
Ineligible for donation	Diagnosis of blood-transmitted disease, medical reason, false-positive result in screening tests
Lack of time because of work	Not enough time for donation
No information about the need for blood	Not knowing about the need for blood, unaware of the need for blood donations

The most frequent reasons for lapsed donation were lack of time because of work and self-exclusion for medical reasons. Among repeat donors, the most frequent reasons for not returning to donate were lack of time because of work and illness. Among first-time donors, the most frequent reasons were ineligibility for donation and lack of time because of work (Table 4). Not returning to donate because of fear of needles, fear of contracting disease or fear of complications from the donation were more frequent among first-time donors (P < 0.05). The reasons for not returning among donors less than 25 years of age were lack of time because of work and ineligibility for donation. Among donors over 25 years of age, the most frequent reasons were lack of time because of work and illness. Not donating because of lack of awareness of the need for blood was most frequent among first-time donors (P < 0.05). Not returning to donate because of fear of needles, fear of contracting disease, fear of complications from donation or ineligibility for donation was more frequent among female donors (P < 0.05)

**Table 4.** Reasons for not returning to donate blood, according to donation characteristics

Reason for not returning	First-time donors (%) No.	Repeat donors (%) No.	All donors (%) No.
Not suitable for donation	(33.5%) 100	(31.5%) 114	(32.4%) 214
Lack of time due to work	(31.3%) 93	(34.2%) 124	(32.9%) 217
Fear	(9.7%) 29	(4.2%) 15	(6.7%) 44
No access to donation center	(8.4%) 25	(8.8%) 32	(8.6%) 57
No information about the need for blood	(8.4%) 25	(3.6%) 13	(5.7%) 38
Long wait to donate	(5.4%) 16	(7.4%) 27	(6.5%) 43
Staff factors	(2%) 6	(4.2%) 15	(3.2%) 21
Travel inconvenience	(1.3%) 4	(1.9%) 7	(1.7%) 11
Previous bad donation experience	(0%) 0	(4.2%) 15	(2.3%) 15

## DISCUSSION

Recruiting first-time donors and maintaining the existing donors are essential to ensure an adequate blood supply.^14^ In spite of the eligibility of a large number of individuals in a given population for donation, only a small proportion of the population donates regularly.^23^ Since the prevalence of transfusion-transmitted disease is lower among repeat donors, increasing the number of repeat donors improves blood safety.^12,15-21^

The reasons for not returning to donate are diverse, and may correlate with demographic factors. Therefore, investigation of the main motivational factors that may create barriers to donation is essential. In this study, the main reason for not returning to donate was lack of time because of work. Adjusting donation hours so that donors can donate after working hours may increase repeat donations. In addition, it may be essential to provide mobile teams to regularly visit workplaces and provide opportunities to donate. This would shorten the donation process so that donors do not perceive it as time-consuming.

Not returning to donate because of fear of needles, fear of contracting diseases or fear of complications from donation were more frequent among female donors. This may be due to the higher frequency of vasovagal reactions among them.^29-36^ It therefore seems that education and reassurance for donors are needed, in order to explain to them that donation does not affect their health.

The rate of repeat donation is lower among donors who have had a reaction,^4,6,9^ and the return rate among donors who have had good experiences is higher.^28^ This suggests that education for the phlebotomy staff is required, so that donors experience less pain. Improved donor care, in order to reduce the frequency of donor reactions, is also essential.

Not donating because of lack of awareness of the need for blood was most frequent among the first-time donors. Informing blood donors about the permanent need for blood products is another strategy that is likely to increase donor recruitment. Staff factors (poor staff skills or poor treatment) were less frequently cited by our participants as a deterrent to repeat donation. This provided evidence of professional behavior and good staff-donor relations at our center. Older donors were more likely not to return because of medical reasons. Not returning because of ineligibility for donation was also more frequent among female donors, which may be due to their higher prevalence of iron deficiency.^37^

### Limitations

This study was conducted in order to ascertain the reasons for a decline in blood donations. This study must be seen within the context of the scale of the survey and may not be representative of the population as a whole. There are many factors that have influenced the declining numbers, some of which may not have been mentioned in this study (including, for instance, recall bias). The author would therefore suggest that additional recruitment programs should be implemented in order to overcome the barriers observed in this study, survey the results and access any changes in donor rates.

## CONCLUSIONS

The reasons for not returning to donate are varied and may correlate with demographic characteristics. In this study, the main reason for not returning was lack of time because of work. Changing donation hours so that donors can donate after work, providing mobile teams at workplaces at regular times and shortening the duration of the donation process may help increase repeat donation rates. Not returning to donate because of fear of needles, fear of contracting diseases or fear of complications from donation were more frequent among female donors. It seems that education and reassurance for donors are needed, in order to explain to them that donation does not affect their health.
